# Frontiers of Wearable Biosensors for Human Health Monitoring

**DOI:** 10.3390/bios13110964

**Published:** 2023-10-31

**Authors:** Xiaojun Xian

**Affiliations:** The Department of Electrical Engineering and Computer Science, Jerome J. Lohr College of Engineering, South Dakota State University, Brookings, SD 57007, USA; xiaojun.xian@sdstate.edu

Wearable biosensors offer noninvasive, real-time, and continuous monitoring of diverse human health data, making them invaluable for remote patient tracking, early diagnosis, and personalized medicine. Breakthroughs in biosensing technologies have made it possible to detect both biophysical parameters, such as heart rate [1,2], body temperature [3,4], blood pressure [5,6,7], and ECG [8,9,10], as well as biochemical parameters, including glucose [11,12], lactate [13,14], cortisol [15,16,17], Na^+^ [18,19], and K^+^ [20,21], within various biofluids (like sweat, tears, saliva, and interstitial fluid) [22]. Biosensors have been seamlessly incorporated into a wide range of wearable platforms, such as contact lenses [23,24], wristbands [25,26], patches [27,28], tattoos [29,30], and retainers [31]. While these advances are promising, there remain challenges in terms of accuracy, stability, multiplexed sensing, energy harvesting, and system integration that require further technical innovations [32]. The potential for commercialization and the anticipated healthcare benefits continue to drive rapid progress in wearable biosensor research.

This Special Issue, “Frontiers of Wearable Biosensors for Human Health Monitoring”, presents ten research articles and three reviews showcasing the latest advancements in wearable biosensing technology development, validation studies, and healthcare applications. The research papers cover a diverse spectrum of wearable biosensor research, ranging from the sensing of electrical impedance, transcutaneous gas, heart rate, capillary oxygen desaturation, ECG waveform, intracranial pressure, intra-abdominal pressure, and sleep patterns to the mitigation of data loss in the wearable healthcare ecosystem. Additionally, the reviews in this Special Issue provide comprehensive insights into flexible wearable sensors in medical monitoring, biosensors based on electromyography (EMG), force myography (FMG), and electrical impedance tomography (EIT), and the utilization of wearables for remote healthcare among the elderly.

Respiratory rate is a fundamental vital sign that offers essential information about a person’s overall health and lung function. Historically, spirometry has been the standard for respiratory rate monitoring, but its bulkiness and complexity pose challenges [33]. Yan et al. introduced a wearable head-mounted system that monitors respiration by measuring electrical impedance in the subpapillary pharynx of the mastoid bone [34]. They successfully simulated impedance changes in the pharynx during breathing, integrated a head-mounted device, and confirmed its effectiveness. Test results showed a high correlation with commercial respiratory monitoring devices, making this system a promising solution for real-time continuous personalized respiratory monitoring in healthcare.

Electrocardiogram (ECG) signals are widely used for diagnosing heart conditions and assessing emotional and psychological states [35]. However, in many wearable ECG systems, only prominent R peaks are evaluated for heart rate variability (HRV) monitoring, while smaller peaks (P, Q, S, and T) are often overlooked. Research indicates that this fine-grained variability data are essential for measuring emotional and stress responses. Arquilla et al. developed various woven textile electrodes (eight different sizes, four different patterns, and two different thread types), testing their performance on 10 participants. They suggest that woven textile electrodes are a viable option for garment-integrated ECG monitoring systems to capture the complete ECG waveform [36]. This research offers valuable guidelines for future woven textile ECG electrode development. Recent research suggests that HRV is an indicator of cognitive fatigue. Continuous monitoring of heart rate through wearable ECG devices is a viable method of assessing cognitive fatigue. However, wearable ECG devices often compromise the sampling rate, affecting HRV signal quality. To explore if lower sampling rates impact HRV features in predicting cognitive fatigue, Lee et al. collected ECG data at 2000 Hz during a typical cognitively fatiguing task, systematically down-sampled the data to various rates, extracted frequency domain features, and developed predictive models [37]. They discovered that a sampling rate of 125 Hz is sufficient for an accurate assessment of cognitive fatigue using frequency domain features. These findings are valuable for designing cost-effective wearables to detect cognitive fatigue.

Bio-pressure measurement plays a vital role in diagnosing various medical conditions. Elevated intracranial pressure (ICP) can be indicative of head injuries, hydrocephalus, or infections [38]. However, monitoring ICP in infants is challenging due to the risks (hemorrhage and infection) associated with invasive techniques and the limitations (low accuracy and high cost) of noninvasive methods. Zhang et al. introduced a novel noninvasive approach using a wearable pressure sensor that measures ICP changes by detecting the electrical resistance variation in a liquid metal (Ga)-filled microchannel when it deforms due to inflation [39]. This innovative ICP sensor, fabricated through a freeze-casting method, can be applied directly to infants like a band-aid. Their results showed a high linear correlation and demonstrated the potential for cost-effective noninvasive ICP monitoring in clinical or point-of-care settings. Intra-abdominal pressure (IAP) is a crucial factor in diagnosing intra-abdominal hypertension (IAH) and abdominal compartment syndrome (ACS) [40]. The existing IAP monitoring method is labor-intensive and provides only a single-point measurement. Kumar et al. introduced a stretchable pressure-sensing sleeve enabling continuous IAP monitoring while significantly reducing the complexity of the process [41]. This sleeve’s sensitivity in low-pressure ranges (<2.7 kPa) is clinically relevant for early IAH and ACS diagnosis. Benchtop testing validated the sleeve’s performance. This stretchable capacitive pressure sensor could potentially reduce the personnel and time needed for continuous IAP monitoring and can be easily adapted with intrabody catheter balloons for on-site intra-abdominal pressure measurement.

Blood oxygen and carbon dioxide levels are essential parameters for evaluating respiratory and metabolic health. Monitoring blood CO_2_ partial pressure (*pCO*_2_) is critical for diagnosing and treating respiratory and metabolic conditions [42]. Instead of invasive arterial blood gas sampling, noninvasive transcutaneous CO_2_ monitoring provides a promising alternative approach [43]. Cascales and colleagues reported the development of a highly breathable CO_2_-sensing film for transcutaneous CO_2_ monitoring through fluorescence quantification [44]. They investigated various HPTS-based ion pairs in diverse support matrices and found the (HPTS)/(TOA)_4_-embedded PPMA matrix was highly sensitive within the physiological CO_2_ range (0–50 mmHg). These CO_2_-sensing films exhibited inherent resistance to humidity variations and maintained photostability during extended continuous sampling. This work holds great potential for commercial, miniaturized wearable devices for transcutaneous CO_2_ monitoring. Obstructive sleep apnea (OSA) is a common sleep disorder [45] for which continuous positive airway pressure (CPAP) is the most effective treatment [46]. Simple, low-cost, at-home diagnostic tools are needed to improve CPAP adherence. Wearable devices, like smartwatches, measure oxygen saturation in arm or wrist tissues. However, it is unclear if arm oxygen desaturation can gauge CPAP effectiveness. Zhang et al. investigated oxygen desaturation in arm muscles using gold-standard frequency domain multi-distance near-infrared spectroscopy (FDMD-NIRS) during CPAP titration in OSA patients [47]. They found only fingertip SpO2, not arm StO2 (muscle tissue oxygen saturation), reflected reduced desaturation during CPAP titration, likely due to the contribution of venous blood to StO2. This suggests that muscular oxygen desaturation may not be an ideal indicator of CPAP effectiveness. This study advises caution in using these wearables until they have been clinically validated.

Wireless communication is a vital feature in wearables, with Bluetooth Low Energy (BLE) being a common choice for data transmission [48,49]. Ensuring reliable and secure data transfer in a wearable healthcare ecosystem requires a systematic evaluation of BLE packet loss and the development of mitigation strategies. Tipparaju et al. conducted a comprehensive assessment of packet losses in Android and iOS-based wearable systems and proposed a mitigation solution that included a reduction in transmission frequency, data bundling, and a queue-based packet transmission protocol [50]. Their approach reduces packet losses to less than 1% and can benefit various applications, such as body sensor networks (BSNs), the Internet of Things (IoT), and smart homes within BLE-based wearable ecosystems.

Machine learning techniques are powerful tools for processing data from wearable sensors, enabling precise disease prediction and early detection [51,52]. In a recent study, Guo et al. developed a 1D-Convolutional Neural Network (CNN) with a Long Short-Term Memory (LSTM)-based evaluation model using self-designed wearable smart bracelets to assess teenagers’ physical fitness [53]. They collected 1024 photoplethysmography (PPG) data from teenagers, applied noise reduction techniques, and constructed a deep learning model to classify physical fitness levels. This deep learning model demonstrates excellent accuracy in predicting physical fitness for both boys and girls. This study highlights the potential of integrating machine learning techniques with wearable devices for well-being prediction.

The articles in this Special Issue demonstrate the latest advancements in wearable biosensor research. Overcoming challenges such as accuracy, stability, selectivity, motion artifacts, power management, regulatory compliance, and commercialization of these biosensors demands further innovative engineering solutions. Moreover, integrating smart materials [54,55], additive manufacturing (3D printing) [56,57,58], artificial intelligence [59,60], the Internet of Things (IoT) [61,62], and big data [63,64] can further enhance the potential of wearable biosensors for human health monitoring.
